# The climatic and genetic heritage of Italian goat breeds with genomic SNP data

**DOI:** 10.1038/s41598-021-89900-2

**Published:** 2021-05-26

**Authors:** Matteo Cortellari, Mario Barbato, Andrea Talenti, Arianna Bionda, Antonello Carta, Roberta Ciampolini, Elena Ciani, Alessandra Crisà, Stefano Frattini, Emiliano Lasagna, Donata Marletta, Salvatore Mastrangelo, Alessio Negro, Ettore Randi, Francesca M. Sarti, Stefano Sartore, Dominga Soglia, Luigi Liotta, Alessandra Stella, Paolo Ajmone-Marsan, Fabio Pilla, Licia Colli, Paola Crepaldi

**Affiliations:** 1 Dipartimento di Scienze Agrarie e Ambientali – Produzione, Territorio, Agroenergia, Università degli Studi di Milano, Via Celoria 2, 20133 Milan, Italy; 2 Dipartimento di Scienze Animali, della Nutrizione e degli Alimenti and BioDNA Centro di ricerca sulla Biodiversità e sul DNA Antico, Università Cattolica del Sacro Cuore, Via Emilia Parmense 84, 29122 Piacenza, Italy; 3 The Roslin Institute, University of Edinburgh, Easter Bush Campus, Midlothian, EH25 9RG UK; 4 Unità di Ricerca di Genetica e Biotecnologie, Agris Sardegna, 07100 Sassari, Italy; 5 Dipartimento di Scienze Veterinarie, Università di Pisa, Viale delle Piagge 2, 56124 Pisa, Italy; 6 Dipartimento di Bioscienze Biotecnologie e Biofarmaceutica, Università degli Studi di Bari, Via Orabona 4, 70126 Bari, Italy; 7 Consiglio per la ricerca in agricoltura e l’analisi dell’economia agraria (CREA) - Research Centre for Animal Production and Acquaculture, 00015 Monterotondo, Rome, Italy; 8 Department of Agricultural, Food and Environmental Sciences, University of Perugia, 06121 Perugia, Italy; 9 Department of Agriculture, Food and Environment, University of Catania, Via Valdisavoia 5, 95123 Catania, Italy; 10 Dipartimento Scienze Agrarie, Alimentari e Forestali, University of Palermo, 90128 Palermo, Italy; 11 Department of Chemistry and Bioscience, Faculty of Engineering and Science, University of Aalborg, Aalborg, Denmark; 12 Dipartimento di Scienze Veterinarie, Università degli Studi di Torino, largo Braccini 2, 10095 Grugliasco, Italy; 13 Dipartimento di Scienze Veterinarie, University of Messina, Messina, Italy; 14 Institute of Biology and Biotechnology in Agriculture, National Research Council (CNR), Milan, Italy; 15 Dipartimento Agricoltura, Ambiente e Alimenti Universitá degli Studi del Molise, 86100 Campobasso, Italy

**Keywords:** Haplotypes, Biodiversity, Genomics, Population genetics

## Abstract

Local adaptation of animals to the environment can abruptly become a burden when faced with rapid climatic changes such as those foreseen for the Italian peninsula over the next 70 years. Our study investigates the genetic structure of the Italian goat populations and links it with the environment and how genetics might evolve over the next 50 years. We used one of the largest national datasets including > 1000 goats from 33 populations across the Italian peninsula collected by the Italian Goat Consortium and genotyped with over 50 k markers. Our results showed that Italian goats can be discriminated in three groups reflective of the Italian geography and its geo-political situation preceding the country unification around two centuries ago. We leveraged the remarkable genetic and geographical diversity of the Italian goat populations and performed landscape genomics analysis to disentangle the relationship between genotype and environment, finding 64 SNPs intercepting genomic regions linked to growth, circadian rhythm, fertility, and inflammatory response. Lastly, we calculated the hypothetical future genotypic frequencies of the most relevant SNPs identified through landscape genomics to evaluate their long-term effect on the genetic structure of the Italian goat populations. Our results provide an insight into the past and the future of the Italian local goat populations, helping the institutions in defining new conservation strategy plans that could preserve their diversity and their link to local realities challenged by climate change.

## Introduction

The preservation of animal genetic diversity is fundamental to ensure food security and the development of farming communities^1^. Among the key factors shaping genetic and phenotypic diversity there is climatic and environmental heterogeneity^2^, with indigenous domestic breeds showing better adaptation to local environments than highly productive breeds kept in controlled farming systems in which the effects of climatic challenges are minimized^3–5^. In this rapidly evolving situation, species that are mostly reared in marginal rural areas of the world such as goats are also more likely to be among those most affected by environmental changes^6^.

The Italian territory is characterized by a rich environmental diversity, spanning from the polar climate of the Alps to the Mediterranean climate of the south and isles^7^. This environmental richness is paired with a reservoir of genetic resources for the caprine species counting over 36 goat breeds registered by the National Goat and Sheep breeds Association (http://www.assonapa.it). Italian goat genetic resources are managed throughout diverse farming systems ranging from intensive and semi-intensive to traditional grazing and transhumance. Importantly, most of them pertain to marginal areas where they play a crucial socio-economic role, contributing to the management of landscapes, biodiversity preservation, and the production of niche traditional products^8^. The Italian caprine diversity and adaptation to climate has been previously investigated through genotype data analyses^6,9^. However, no previous work included breeds from the central areas of the country, which hosts several minor, local, and niche populations^10^. Importantly, the latter are specifically adapted to the broad range of Italian eco-climatic scenarios, making them an ideal model to investigate genetic adaptation to climate, which only few studies tried to address in the goat species^11–13^.To investigate the link between territory and genetics, we analyse the second release of the goat genotype dataset assembled by the Italian Goat Consortium, a collaboration across Italian universities that aims to enhance the understanding of the Italian goat genetic variability. This new release of the dataset, called IGC2, expands the previous version, improving the sampling coverage of the central-southern goat populations and completes the whole Italian panorama and its internal connections (Supplementary Fig. 1). To the best of our knowledge, IGC2 currently represents the largest national-level genotyping effort on goat biodiversity^10^.

The most recent climate predictions by the Koppen-Geiger climate classification foresee hotter and drier climate across the Italian peninsula over the next 70 years^14^. Such changes will likely affect locally adapted populations by reducing food availability (e.g., pasture and forage crop availability and quality^15^), increase temperature-related health problems (illness, death rates, increased diffusion of vector-borne diseases and parasites), and cause metabolic problems (decreasing productive and reproductive performance or depressing feed intake^8,16^). Locally adapted breeds will be the hardest hit, mostly relying on grazing^15,17^.

We performed genome-wide analyses on the IGC2 dataset to investigate the genetic structure of Italian goat breeds with particular attention to the newly sampled local populations, and link the heritage and genomic structure of current populations with the present and future climatic condition of the rearing areas. Our results will help to understand their environment-driven adaptation and draw effective management plans to face climate change^18,19^.

## Results and discussion

### Genotyping control and datasets creations

After filtering the initial raw dataset of 1,071 goats for poor quality genotype and related animals we obtained the dataset used for the haplotype sharing analysis, which included 980 animals and 48,396 SNPs. This dataset was further pruned for linkage disequilibrium (LD; r^2^ < 0.2).

We first balanced the number of animals in the different populations by reducing the size of the nine largest groups, leaving 42,088 SNPs and 802 individuals. We used this dataset to perform population structure analyses (Multi-Dimensional Scaling (MDS), Admixture, and Reynolds distances).

Upon the removal of 2nd-degree related individuals and animals without geographical coordinates, we retained 41,898 SNPs and 489 individuals for Landscape Genomics analyses. See Table 1 for the detailing of the different datasets.

**Table 1:** Composition of the datasets used for the different analysis, with names, codes and number of samples processed and filtered for each population and grouped by the type of analysis.

Breeds ID	Breeds name	Raw dataset (n animals)	Haplotype sharing (n animals)	Population structure (n animals)	Landscape Genomics (n animals)
ALP	Camosciata delle Alpi	143	117	30	43
ARG	Argentata dell'Etna	48	46	30	41
ASP	Capra dell'Aspromonte	24	24	24	18
BEZ	Bezoar (outgroup)	7	7	7	0
BIA	Bianca Monticellana	24	23	23	17
BIO	Bionda dell'Adamello	24	24	24	22
CAP	Capestrina	24	22	22	15
DDS	Derivata di Siria	32	25	25	11
FAC	Facciuta della Valnerina	24	24	24	12
FUL	Fulva del Lazio	22	20	20	14
GAR	Garganica	40	37	30	3
GCI	Grigia Ciociara	43	39	30	18
GIR	Girgentana	59	56	30	9
GRF	Garfagnana	28	25	25	18
JON	Jonica	16	15	15	3
LIV	Capra di Livo	24	22	22	19
MAL	Maltese	16	16	16	10
MES	Messinese	24	23	23	21
MNT_M	Montecristo (mainland)	18	12	12	1
MNT_I	Montecristo (island)	24	23	23	1
MON	Capra di Montefalcone	24	23	23	13
MXS	Incrocio Maltese e Sarda	36	36	30	0
NIC	Nicastrese	24	24	24	14
NVE	Nera di Verzasca	19	19	19	11
ORO	Orobica	23	23	23	5
RCC	Roccaverano	28	28	28	25
RME	Rossa Mediterranea	46	40	30	13
SAA	Saanen	44	44	30	20
SAM	Maltese sampled in Sardinia	15	15	15	5
SAR	Sarda	33	33	30	32
TER	Capra di Teramo	43	30	30	8
VAL	Valdostana	24	24	24	16
VLS	Vallesana	24	17	17	7
VPS	Capra della Val Passiria	24	24	24	24
TOTAL Animals		1071	980	802	489

### Population structure

**Figure 1:**
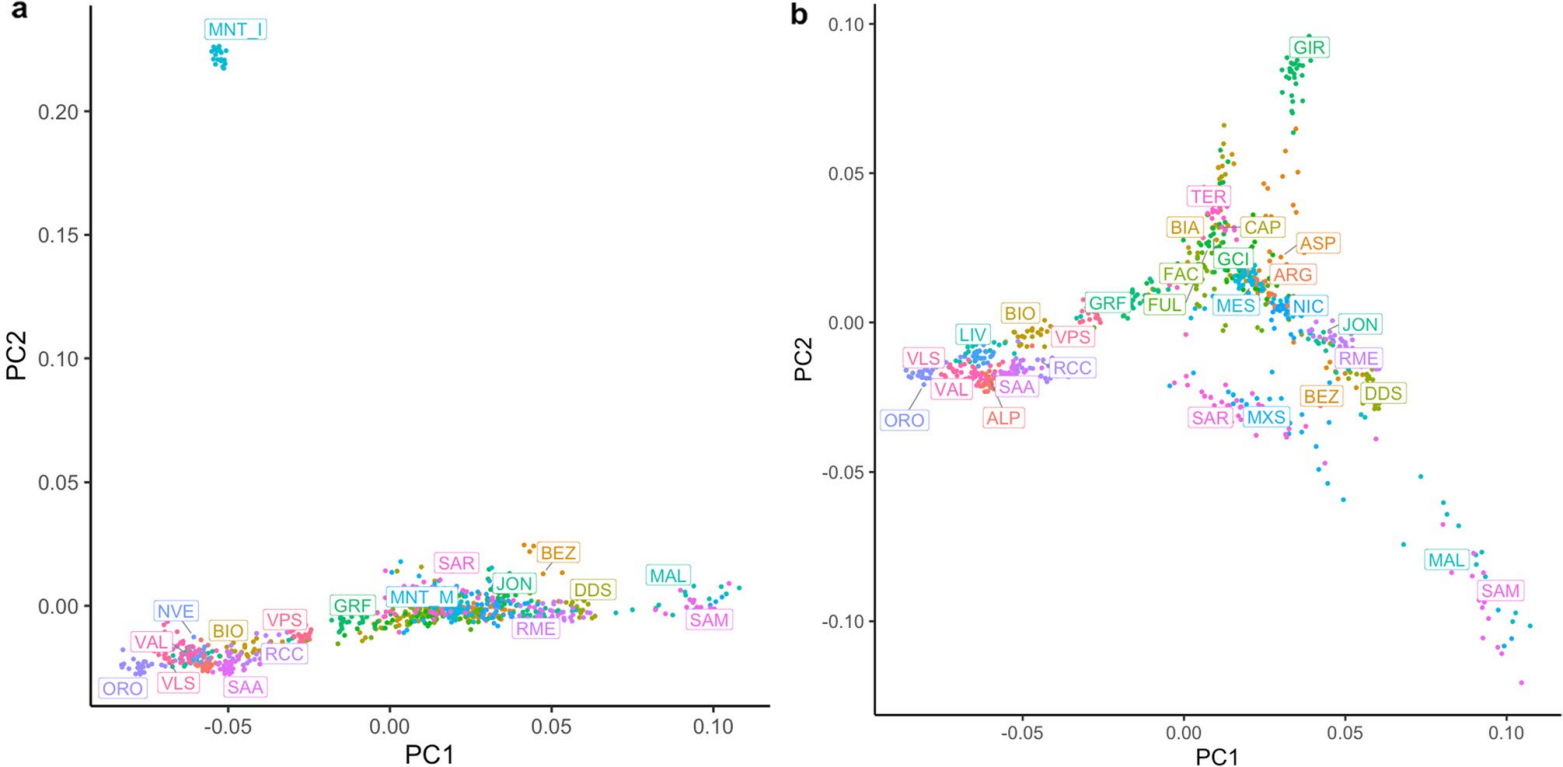
(**a**) MDS plot representing all the populations contained in the dataset, (**b**) MDS plot without the two Montecristo populations (MNT_M e MNT_I).

The MDS plot showed a north–south geographic gradient comparable with previous findings on Italian goat population structure^6^. The first MDS component identified three main groups corresponding to northern Italian, central-southern Italian, and Maltese populations. The second MDS component discriminated the insular Montecristo goat (MNT_I; Fig. 1a) from the other mainland breeds, likely due to the high inbreeding and prolonged geographical isolation (Somenzi et al., in preparation). For this reason, we excluded the two Montecristo populations (MNT_M and MNT_I) from the subsequent population structure and haplotype sharing analysis and to repeat the analyses without them. The new MDS plot without the two MNT populations (Fig. 1b) still separated the three main groups on the first component, a structure further supported by the bootstrapped Reynolds’ distances phylogenetic tree (Supplementary Fig. 2).

**Figure 2:**
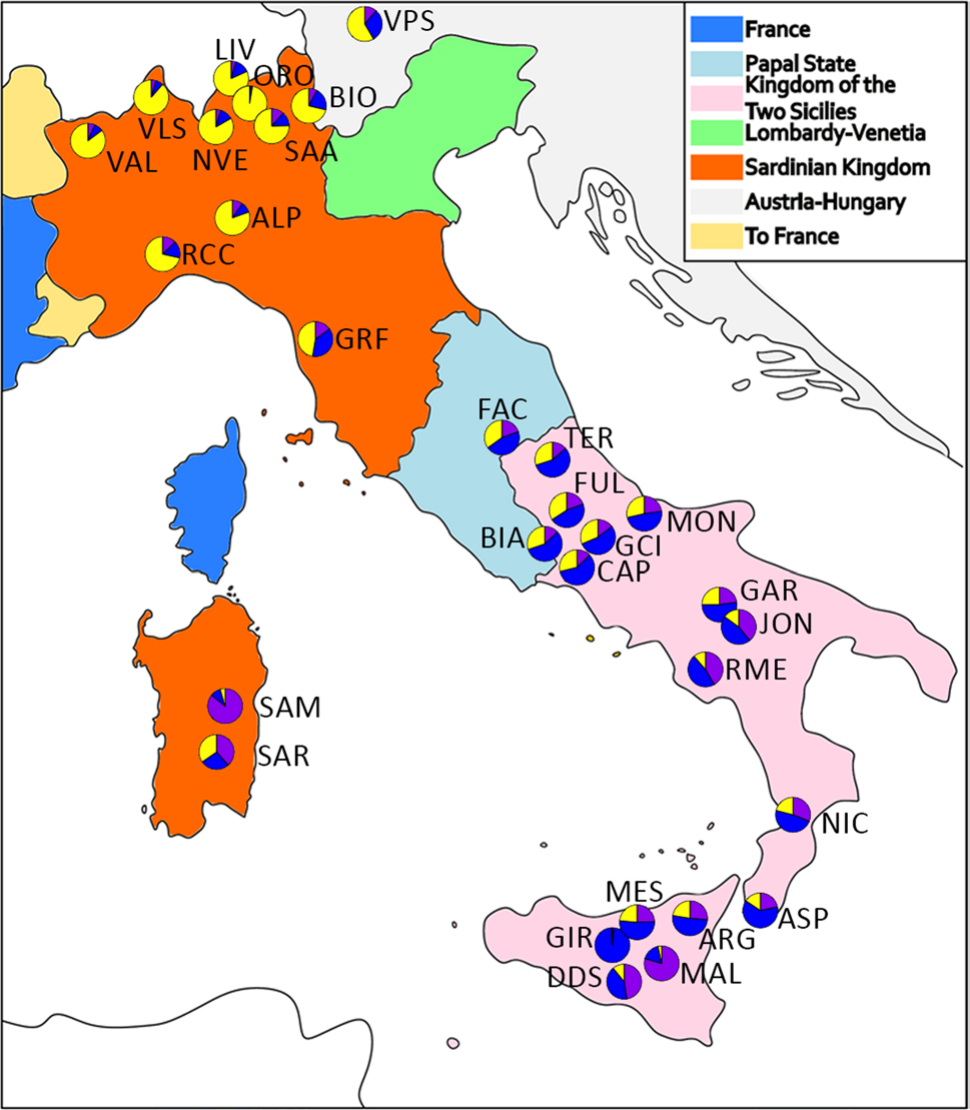
Historical map of Italy pre-unification with pie chart of the ADMIXTURE *K* = 3 values plotted on the geographical coordinates of the mean sampling location of each breed. The colours reflect the ADMIXTURE component associated with the Maltese (purple), Northern Italy Cluster (yellow), and Central-Southern Cluster (blue). BEZ and MXS were not represented due to the lack of specific geographic coordinates. MNT_I and MNT_M were excluded as extreme outliers. Map was generated in Inkscape v1.0 (https://inkscape.org/); the pie charts were created using R^21^.

Although overlapping a previous investigation of the Italian caprine population structure^6^, our improved dataset identified a closer relationship between the central and southern Italian population, more in accordance with the recent known history and geography of the Country. Until 1860 Italy was divided in many states with tight connections to other European kingdoms (https://www.150anni.it). The north-western part of the country and Sardinia were part of the Sardinian kingdom, tightly connected with the French empire, whereas the north-east part (the Kingdom of Lombardy—Venetia) was under the political influence of the Austrian Empire. Central Italy was ruled by the Papal state, and southern Italy and Sicily were under the Kingdom of the two Sicilies ruled by the Borbone (Fig. 2)^20^.

The ADMIXTURE analysis (Supplementary Fig. 3) at *K* = 2 separates the Maltese populations (purple component) and the Northern Italy breeds (yellow component), and improves the representation of the North–South gradient over previous studies on Italian goat populations^6^. At *K* = 3 it resembles the MDS plot distinguishing the central-southern Italian breeds led by the Girgentana (GIR; blue component) and the mean proportion for each breed overlap nicely with the political borders of Italy prior 1860 (Fig. 2). Each *K* above 3 distinguishes single or groups of breeds, such as Teramana (TER; *K* = 4) and Valdostana (VAL; *K* = 5). The lowest cross-validation error was recorded at *K* = 20 (Supplementary Fig. 4) and showed the similar genetic background of those breeds originated from the same geographical regions (north, central, south and Maltese), and some breeds identified by private clusters, once again confirming the uniqueness of GIR, ORO, VAL, TER and SAM, among others (Supplementary Fig. 3).

**Figure 3:**
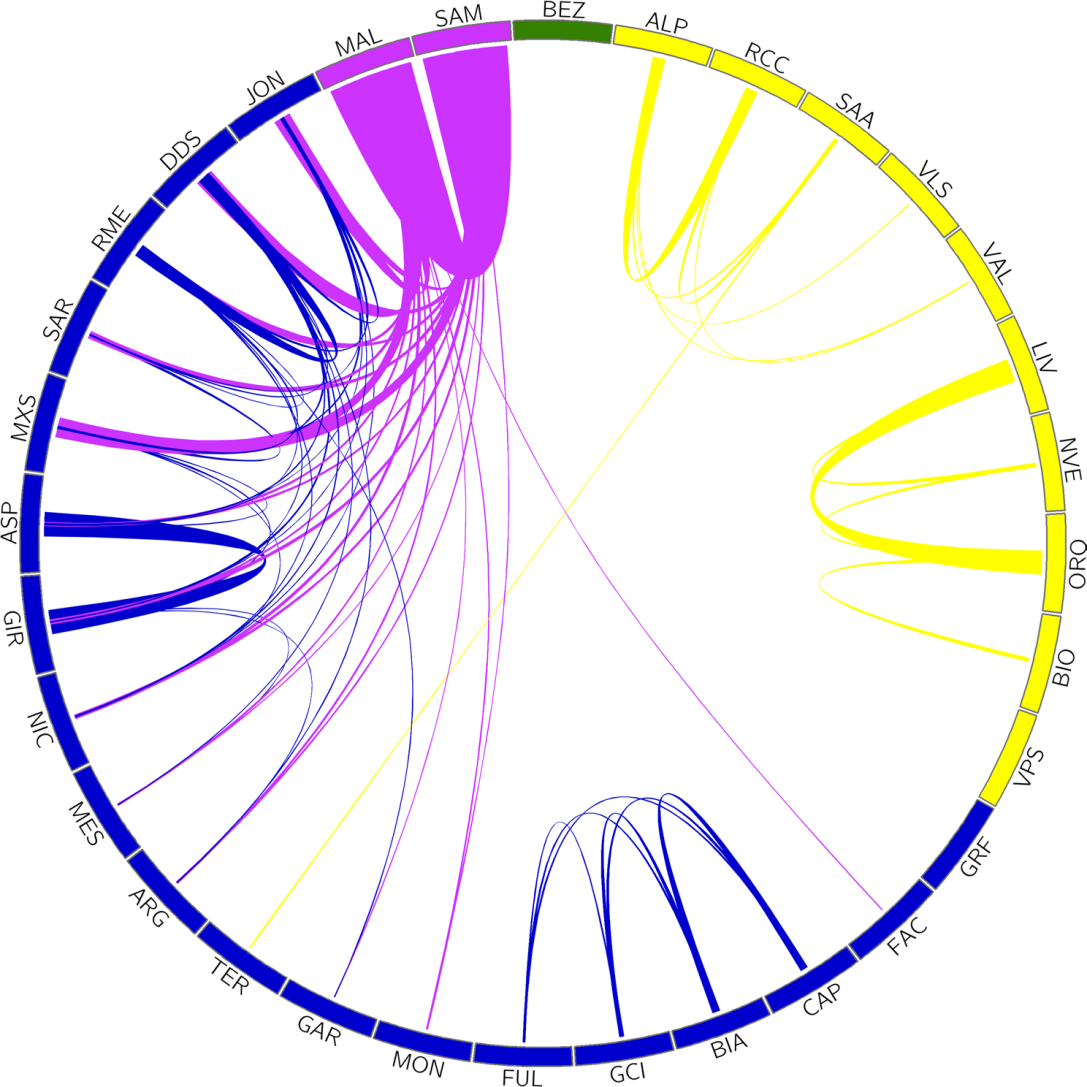
Proportion of the median haplotype shared among the Italian goat populations. The colours reflect the ADMIXTURE components at *K* = 3, which overlap with the administrative and temporal history of the Italian Peninsula until 1860. The outgroup is highlighted in green (extended name reported in Table 1). The figure was generated using Circos v0.69-8 Software^22^.

The haplotype sharing analysis across populations (Fig. 3) also highlights the three genetic groups corresponding to admixture *K* = 3 and consistently with the administrative and temporal history of the Italian Peninsula until 1860^20^.

We observe that the Northern-Italian populations (yellow cluster) show no haplotype exchange with the other clusters, with the exception of SAA and TER probably due to a recent introgression event. Within the Northern-Italian cluster there is a more pronounced haplotype sharing among the Lombardy breeds (ORO, NVE, LIV and BIO) than among those from the rest of the Alps. The Val Passiria (VPS) together with the Garfagnana (GAR) are the only two populations that do not exchange haplotypes at all, perhaps suggesting a geographical and/or political isolation. Populations from Central-Southern Italy (blue cluster) show large haplotype sharing within and among different clusters, possibly due to breeding and management practices as well as local geographical conditions, such as breeds from the Lazio region (BIA, GCI, CAP, and FUL) have high haplotype sharing among themselves. Lastly, the populations from the isles and in particular the Maltese (MAL and SAM, purple cluster) and Sarda (SAR and MXS) are those that mostly shared haplotypes with all other southern breeds, probably as a consequence of their high productivity and diffusion over the territory. The green colour represents the outgroup *Capra aegagrus* that does not exchange haplotypes with any of the other breeds. Importantly, future investigations with dedicated experimental designs aimed to dissect the different effects of selection might aid unfolding the undergoing evolutionary dynamics.

The political subdivision of Italy preceding the unification of the country has probably contributed to maintain the ancient genetic flows from central-north Europe in the north of the country and from Africa and Spain in the south^13^, with only a minor impact on the population structure of the following 150 years of history of the country.

### Landscape genomics

The landscape genomics analyses (LGA) were performed using the climatic variables representing the current climate applying two different approaches: Samβada^23^ and LFMM^24^. We observed no direct overlap between the two methods. However, this is not surprising as simulation studies showed that LFMM is overall more conservative than Samβada, and the two methods tend to have marginal overlap on co-selecting the same signals, with the most significant loci detected by Samβada ignored by LFMM^23^. Samβada identified 252 genotypes belonging to 216 different SNPs significantly associated (FDR < 0.05) with at least one climatic variable (Supplementary Table 1). Among them, 75 SNPs mapped within a gene region annotated in the goat genome (ARS1.2), identifying a total of 62 different genes associated with at least one of the following four representative environmental variables: “Isothermality” (47 genes), “Mean diurnal range” (four genes), “Mean temperature wettest quarter” (three genes) and “Precipitation coldest Quarter” (11 genes) (Supplementary Table 2). Some of these genes had already been identified in other landscape genomics works in relation with different environmental variables, for example ANK3 and BTRC in relation to longitude, and RYR3 with Mean Temperature of the wettest quarter (BIO3)^19^. The DCLK1 gene, in particular, was found in association with the continental goat group compared to the rest of the world^9^. Details on correlations among representative and excluded variables are shown in Supplementary Table 3.

Initially, we investigated the role in biological pathways of the 62 genes identified by Samβada (Supplementary Table 2), splitting them according to the associated environmental variable. We identified only one significant pathway (“Circadian rhythm related genes WP3594”; adjusted *p*-value < 0.0045) for those genes associated with “Mean diurnal range” with two genes linked to the circadian clock regulation (*MAPK9*^25^) and to hair follicle formation and hair growth in Cashmere goat (*NTKR3*^26^).

We also analysed the 62 genes individually to better understand their function. Using the information found, we can clump the most interesting genes into four groups based on the phenotype they affect the most: (1) meat- and growth-related genes, (2) circadian rhythm-related genes, (3) fertility-related genes, and (4) inflammatory response genes.

The first group (meat and growth) is the largest and counts 24 genes, including *HADC9*, which has a role in the feedback inhibition of myogenic differentiation in sheep muscle^27^, *DLG1*, that is related to adipogenesis and residual feed intake in cows^28^, and *KLF12*, which is related to the formation of preadipocytes in goats^29^. The second group (circadian rhythm) includes 12 genes, such as *MAPK9* and *EYA3*, both related to melanin production and photoperiod regulation^30^, and *KCNJ1*, associated with the production of polyunsaturated fatty acid (PUFA) and feed efficiency in cattle^5,31,32^. The third group (fertility-related) includes 15 genes such as *BTRC*, whose mutations can affect spermatogenesis and mammary gland development in mice^33^, *PRKD1*, associated to age at puberty in pigs^34^, and *DENND1A*, related to anti-Mullerian hormone and superovulation in dairy cows and to polycystic ovarian syndrome in human^35^. Finally, the fourth group (inflammatory response) includes eight genes such as *BTLA*, strongly related to rheumatoid arthritis^36^. This last gene in particular is relevant as a candidate for one of the most relevant infective diseases of goats worldwide, the Caprine Arthritis Encephalitis Virus (CAEV). This virus belongs to the *Retroviriade* virus family, like the human immunodeficiency virus (HIV), and has rheumatoid arthritis among its principal symptoms^37,38^. Due to the CAEV importance and the relevance of climatic factors and their change play into pathogens diffusion^39^, this group of genes becomes a potential candidate for studies on livestock resilience to incoming climate challenges.

LFMM identified four SNPs significantly associated (FDR < 0.05) with three different climatic variables (Mean Diurnal Range, Mean Temperature Wettest Quarter, and SlopeP), two of which intercepting NBEA, a gene located within a region involved with wool production in sheep^40^ and previously associated with continental goat group in the work of Bertolini et all 2018^9^, and the RHOBTB1 gene that is known to be associated to meat quality in cattle^41^ (Supplementary Table 4).

### Future genotypes prediction

**Figure 4:**
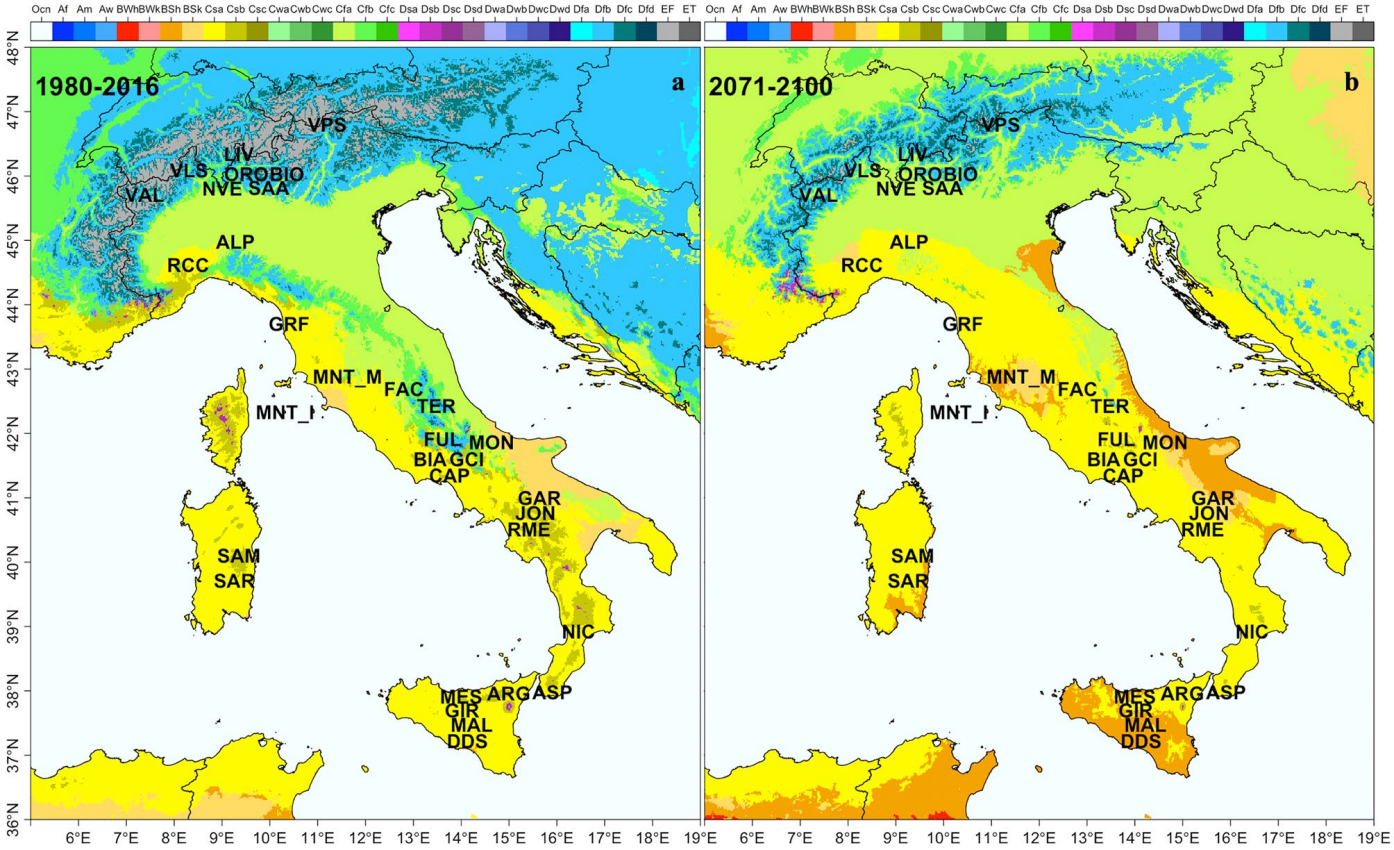
Koppen-Geiger climate maps with classification for Italy (**a**) present day (**b**) future (extended name reported in Table 1). The Maps were generated using R combining the information available in Rubel et al.^42^ and Beck et al.^14^.

The data collected on the current Koppen-Geiger climate classification showed that 21 Italian breeds live in “Temperate” regions, eight in “Cold” regions (BIO, SAA, VLS, TER, MNT_M, LIV, ORO, VPS), two in “Arid” regions (GAR, MNT_I), and one in a “Polar” region (VAL; Fig. 4a). BEZ and MXS were not considered for the analysis due to lack of georeferenced information. If we compare the current Koppen-Geiger classification of their breeding areas with the future predictions (Fig. 4b), we observe that, in 70 years from now, only 11 breeds will live in regions that will not change their classification. Such a scenario will likely pose new threats to those populations living in colder climates, whereas those breeds coming from the warmer parts of the country might have a chance to expand their range, with direct repercussions on the genetic diversity and survival of these breeds.

Among them, nine (ASP, BIA, CAP, GRF, MES, NIC, RCC, RME, SAR) populate “Temperate dry hot summer (Csa)” areas, one (GAR) is present in an “Arid step cold (Bsk)” area, and one (NVE) in a “Temperate without dry season hot summer (Cfa)” region. The remaining 21 breeds populate regions with a warmer and drier climate in the future (Table 2).

**Table 2:** Present and future predicted Koppen-climate class and Anova classification for breed divided in the two groups: HOT/NOTHOT and DRY/NOTDRY (see Materials and Methods).

Breeds	Current class	Future class	Hot-Nothot	Dry-Notdry
GAR	BSk-arid steppe cold	BSk-arid steppe cold	Nothot	Dry
**MNT_I**	BSk-arid steppe cold	Csa-temperate dry hot summer	Nothot	Dry
**MON**	Cfa-temperate without dry season hot summer	BSk-arid steppe cold	Hot	Notdry
NVE	Cfa-temperate without dry season hot summer	Cfa-temperate without dry season hot summer	Hot	Notdry
**ALP**	Cfa-temperate without dry season hot summer	Csa-temperate dry hot summer	Hot	Notdry
**FAC**	Cfa-temperate without dry season hot summer	Csa-temperate dry hot summer	Hot	Notdry
**FUL**	Cfb-temperate without dry season warm summer	Csa-temperate dry hot summer	Nothot	Notdry
**DDS**	Csa-temperate dry hot summer	BSh-arid steppe hot	Hot	Dry
**GIR**	Csa-temperate dry hot summer	BSh-arid steppe hot	Hot	Dry
**MAL**	Csa-temperate dry hot summer	BSh-arid steppe hot	Hot	Dry
ASP	Csa-temperate dry hot summer	Csa-temperate dry hot summer	Hot	Dry
BIA	Csa-temperate dry hot summer	Csa-temperate dry hot summer	Hot	Dry
CAP	Csa-temperate dry hot summer	Csa-temperate dry hot summer	Hot	Dry
GRF	Csa-temperate dry hot summer	Csa-temperate dry hot summer	Hot	Dry
MES	Csa-temperate dry hot summer	Csa-temperate dry hot summer	Hot	Dry
NIC	Csa-temperate dry hot summer	Csa-temperate dry hot summer	Hot	Dry
RCC	Csa-temperate dry hot summer	Csa-temperate dry hot summer	Hot	Dry
RME	Csa-temperate dry hot summer	Csa-temperate dry hot summer	Hot	Dry
SAR	Csa-temperate dry hot summer	Csa-temperate dry hot summer	Hot	Dry
**ARG**	Csb-temperate dry warm summer	Csa-temperate dry hot summer	Nothot	Dry
**GCI**	Csb-temperate dry warm summer	Csa-temperate dry hot summer	Nothot	Dry
**JON**	Csb-temperate dry warm summer	Csa-temperate dry hot summer	Nothot	Dry
**SAM**	Csb-temperate dry warm summer	Csa-temperate dry hot summer	Nothot	Dry
**BIO**	Dfb-cold without dry season warm summer	Cfa-temperate without dry season hot summer	Nothot	Notdry
**SAA**	Dfb-cold without dry season warm summer	Cfa-temperate without dry season hot summer	Nothot	Notdry
**VLS**	Dfb-cold without dry season warm summer	Cfa-temperate without dry season hot summer	Nothot	Nodry
TER	Dfb-cold without dry season warm summer	Csa-temperate dry hot summer	Nothot	Notdry
MNT_M	Dfb-cold without dry season warm summer	Csb-temperate dry warm summer	Nothot	Notdry
**LIV**	Dfc-cold without dry season cold summer	Dfb-cold without dry season warm summer	Nothot	Notdry
**ORO**	Dfc-cold without dry season cold summer	Dfb-cold without dry season warm summer	Nothot	Notdry
**VPS**	Dfc-cold without dry season cold summer	Dfb-cold without dry season warm summer	Nothot	Notdry
**VAL**	EF-polar tundra	Dfb-cold without dry season warm summer	Nothot	Notdry

In bold those breeds that will change their Koppen classification in the next 70 years.

A one-way ANOVA analysis applied on the groups based on the Koppen-Geiger classification identified 27 SNPs that significantly differentiate the groups DRY/NOTDRY (seven within a gene region) and 11 that differentiate the groups HOT/NOTHOT (two within a gene region) (Supplementary Table 5). The linear regression model, applied to verify the variation of the genotype frequencies over time based on the value of their related variables, allowed us to identify five significant SNPs out of nine, intercepting the genes *CHD2, ARL13B, KLF12*, and *PAK5* for the DRY/NOTDRY group and *RACGAP1* for the HOT/NOTHOT group (Supplementary Table 6). Then, we calculated the expected future variation of allelic and genotypic frequencies of the significant SNPs in these groups. For instance, the SNP “snp32991-scaffold385-133908” intercepts the *ARL13B* gene and is associated to “Isothermality” with the genotype GG. At present, the frequency of the G allele of this SNP is 0.4296 in the DRY group and 0.6109 in the NOTDRY group and the delta of the variable “Isothermality” for the two groups is respectively − 0.1253 for the DRY group and − 0.0935 for the NOTDRY group. Using the regressor of the linear regression model (b = 0.3278), we predicted the future G allele frequency for this SNP in both groups (0.3885 and 0.5802 for the DRY and NOTDRY group, respectively) and consequently the expected GG genotype frequency (respectively 0.1509 for the DRY group and 0.3366 in the NOTDRY group). This simplified model suggests a future reduction of the genotype currently associated with the reference variable (“Isothermality”) in both groups. Interestingly, the gene intercepted by *ARL13B* interacts with *RABGEF1*, related to the reduction of the circadian cycle in humans according to the GenomeRNAi human phenotypes database (http://www.genomernai.org). In general, the prediction analysis identified SNPs that might go to stabilization of the frequencies or fixation (see “snp44855-scaffold611-263638” and “snp40739-scaffold521-1667886”, respectively; Table 3).

**Table 3:** Predicted genotypic frequencies for the five polymorphisms recorded within genes identified to be significantly different between the groups HOT/NOTHOT or DRY/NOTDRY.

SNP	Gene	Genotype	Group	AA current	GG current	AG current	AA future	GG future	AG future
snp32991-scaffold385-133908	ARL13b	GG	DRY	0.33	0.18	0.49	0.37	0.15	0.48
			NOTDRY	0.15	0.37	0.48	0.18	0.34	0.49
snp35938-scaffold431-1169604	KLF12	AA,AG	DRY	0.81	0.01	0.18	0.75	0.02	0.23
			NOTDRY	0.64	0.04	0.32	0.60	0.05	0.35
snp23847-scaffold240-2578654	CHD2	AA	DRY	0.59	0.05	0.36	0.54	0.07	0.39
			NOTDRY	0.36	0.16	0.48	0.33	0.18	0.49
snp44855-scaffold611-263638	PAK5	AG	DRY	0.01	0.84	0.15	0.01	0.80	0.19
			NOTDRY	0.02	0.75	0.23	0.03	0.68	0.29
snp40739-scaffold521-1667886	RACGAP1	GG	HOT	0.06	0.56	0.37	0.05	0.61	0.34
			NOTHOT	0.02	0.73	0.25	0.02	0.77	0.21

## Conclusions

This new release of the Italian goat consortium dataset (IGC2)—almost three times the size of the previous iteration—fills in the gaps in terms of completeness and representativeness of the Italian caprine diversity. Our analyses overlap and expand on previous studies providing insight into the past, present, and future evolution of the populations considered. We confirm the geographic gradient of goat diversity ranging from north to south^6^, provide fine scale population structure, and highlight the overlap with the geo-political situation in which the breeds evolved. Previous studies have shown how past migrations from Africa and Spain on the one hand, and central Europe and the Alps on the other hand, contributed to shaping the backbone of biodiversity along the peninsula. Nevertheless, the overlap among the three diversity clusters and the political subdivision of Italy up to 160 years ago^20^ is an intriguing finding that suggests a role for the past socio-political scenario of the country in the current diversity of Italian goats breeds. By investigating the relationship between genotype and environment, we identified several genes which might play a role in the adaptation to temperature and humidity. Interestingly, we identified a gene that can be a fitting candidate for future studies on the caprine arthritis encephalitis virus (CAEV). Lastly, we predicted the future genotypic frequencies under the light of climate changes and foresee the directionality of changes in genotypes frequencies, an important starting point for future studies aiming at improving these analytical approaches. We infer that improved modelling approaches could deepen and perfect such results and shed light on today’s favorable genotypes for specific environmental conditions. These results will likely be instrumental in breeding schemes and genomic selection, assisting locally adapted breeds to cope with the expected climate change toward warmer and drier climates^14^.

## Material and methods

### Biological samples

Management and handling of the animals involved in this study were performed following the Italian and European legislation on animal welfare (D.lgs n. 146/2001, Council Directive 98/58/CE) and adhering to the ARRIVE ESSENTIAL 10 guidelines, where applicable. Blood samples were taken by official veterinary surgeons following the recommendations of the European directive 2010/63, without performing any actual experimental research on animals. Experimental protocol was approved by the Ethical committee of the Department of Veterinary Science of the University of Messina (code 046/2020).

Blood sampling collection of the new individuals was performed using Vacutainer tubes with the K-EDTA anticoagulant, then all the samples were stored at − 20 °C until genomic DNA was extracted using a commercial kit (NucleoSpin Blood, Macherey–Nagel, GmbH & Co KG, Germany) according to the manufacturer’s instructions^6^. DNA samples were genotyped using the GoatSNP50 BeadChip (Illumina Inc., San Diego, CA) developed by the International Goat Genome Consortium (IGGC) at the Agrotis srl (http://www.lgscr.it, Cremona, Italy), Porto Conte Ricerche s.r.l. (Alghero, Sassari, Italy), and University of Palermo facilities (Italy).

### Genotyping control and datasets creations

The IGC2 successfully fills the gaps of the previous dataset^6^, intercepting the local diversity of several under-represented areas of the country (i.e., the central regions of Italy) and identifying small, indigenous breeds never characterized before. For this work, 19 new Italian goat populations, for a total amount of 586 individuals, were sampled and added to SNP genotyping data taken from previously studies^6,43,44^, including seven Iranian Bezoar (*Capra aegagrus*) genotyped by the NEXTGEN project as outgroup for the analyses (“NEXTGEN Project” n.d.). From that, we obtained a final raw dataset consisting of 1071 goats from 33 Italian breeds and populations and one wild species, *Capra aegagrus* (Table 1). Geographical coordinates of the samples at the time of sampling were available for 998 samples (93% of all samples).

The raw dataset was updated to the latest goat genome map (ARS1.2) and pruned to retain SNPs having SNP call rate > 98%, individual call rate > 95% and minor allele frequency (MAF) > 0.05. Then, we pruned loci in linkage disequilibrium (LD), removing one of each couple of SNPs having LD > 0.2 using PLINK v1.90b6^45^. Duplicated individuals (identity by state > 99%) were removed and for each pair of highly related animals (Mendelian Errors count < 100) we excluded the animal occurring in multiple pairs or having the highest missingness. Phasing and imputation of missing genotypes was performed using BEAGLE v4.1^46^, using sliding windows of ~ 5 Mb with an overlap of ~ 2 Mb and allowing two SNPs trimming (~ 0.15 Mb). The resulting dataset was used for the haplotype sharing analysis. In order to investigate the population structure using comparable population sizes, we created a specific dataset reducing the number of individuals for each population to ≤ 30 while maintaining the overall within-population diversity, using the ‘representative.sample’ function implemented in the R package BITE v1.2.0007^47^.

Lastly, individuals with more than second-degree relatedness were identified using the—genome flag in PLINK and removed to perform the Landscape genomics analisis.

### Population structure analysis

Population structure analysis was conducted through MDS of the identity by state (IBS) distances obtained with the flag—cluster in PLINK. Maximum likelihood analysis of population structure was conducted using ADMIXTURE v1.3.0 Software^48^. Unsupervised clustering was calculated for *K* values from 2 to 35. We used fivefold cross-validation (CV) errors for each *K* to evaluate the optimal partitioning, and plots for each *K* were generated using an in-house R script. A phylogeny tree based on Reynolds Genetic distances, with 100 bootstrap replicates, was computed using a custom script. A Neighbour‐joining consensus tree was generated using PHYLIP v3.697^49^ and using Bezoar as an outgroup.

The proportion of haplotypes shared among breeds was determined as Identity By Descent (IBD) estimation among individuals, and calculated using RefinedIBD v4.1^50^ on all the individuals that passed the initial quality check. Sliding windows size was set to of 1 Mb, reporting windows of at least 0.2 Mb and allowing 0.05 Mb overlap. We considered the shared haplotypes between two breeds as the median length of shared haplotypes among all the possible pairs of individuals belonging to the breeds considered (individual pairs with no haplotype sharing were assigned length = 0^51^).

### Landscape genomics

For each georeferenced sample, we used the ‘extract’ function from the R package raster^52^ to retrieve the values of 19 bioclimatic and elevation variables available from the WorldClim database^53^ as those referring to the time span between 1960 and 1990 as proxy for the current climate, and the estimated future values for 2070 (average for 2061–2080) (Supplementary Table 7). Altitude was used to compute terrain slope through the function raster::terrain. Each variable was retrieved as a raster layer with a spatial resolution of 2.5 arcminutes (~ 4 km). Pairwise correlations were calculated among the climatic variables using JMP^54^.

LGA was performed to assess the genotype/environmental variable association using Samβada v.0.5.3^23^ and LFMM v3.1.2^24^. Analyses were performed using the ‘current’ bioclimatic variables and a more stringent subset of animals. To spare computation time, the number of environmental variables was reduced iteratively by randomly removing one of the two most correlated variables until the maximum correlation across all variables was lower than |r^2^|< 0.7 as implemented in the R function ‘caret::findCorrelation’^55^. To reduce the risk of false positive detections, we evaluated the genetic structure of our dataset through principal component analysis, used the scree plot to identify the number of principal components to keep to adequately describe the dataset, and included the selected PCs as population structure predictors for the association analysis^56,57^. A likelihood-ratio test comparing a null and an alternative model was carried out for each genotype. Specifically, null models included the population structure predictors alone, whereas alternative models included population structure predictors and the focal environmental variable. A genotype was considered significantly associated with the environmental variable if the resulting *p*-value associated with the likelihood-ratio test statistic was lower than the nominal significance threshold of 0.05 after Benjamini-Hochberg (BH) correction for multiple testing. The R function ‘p.adjust’ was used to perform corrections for multiple testing^21^.

### Gene-level analysis

To further investigate the biology underlying the signals identified, we screened all the SNPs that resulted significantly associated with a WorldClim variable for annotated genes of interest at the exact location of each marker in the ARS1.2 goat genome version (https://www.ensembl.org/biomart). These genes were investigated individually (https://www.genecards.org) and used as input for an enrichment analysis for pathways and ontologies using the online tool Enricher (https://amp.pharm.mssm.edu/Enrichr/).

### Future genotypes prediction

Comparing the LGA results with the Koppen-Geiger classification relative to the breeding areas of the different Italian breeds, we tried to predict the future frequencies of the genotypes significantly associated with one or more of the environmental variables considered. First, we estimated the extent of climate change that Italian breeds will face comparing the present and future Koppen-Geiger classification^14^ (Supplementary Table 8). Then, we grouped the breeds based on temperature according to the current Koppen-Geiger classification, creating the HOT and NOTHOT groups, and humidity, creating the DRY and NOTDRY groups. The HOT group included those breeds that live in Csa and Dfa regions and the NOTHOT group included breeds that live in Csb, Dfb, Dfc, Bsk, and EF regions. The DRY group included those breeds that live in Csa, Csb, and Bsk regions and the NOTDRY group included those breeds that live in Cfa, Dfb, Dfc, and EF regions (see Table 2 and Supplementary Table 8 for the detail of the environmental codes).

We calculated the MAF of all the significant SNPs from LGA in each Italian breed using a custom script. We summarized the MAF in each of the four groups and performed a one-way ANOVA analysis using the R base package^21^ considering the HOT/NOTHOT groups or the DRY/NOTDRY groups as source of variation, identifying the SNPs significantly different in the two couples of groups. We applied a linear regression model (R base Package) only on those SNPs that fall within genomic regions including an annotated gene considering the mean allelic frequencies of the SNP and the mean value of the environmental variable resulted significantly associated with that SNP for each breed in each group. Finally, we calculated the future hypothetical change in allelic and genotypic frequencies only for those SNPs with a statistically significant linear regression model. For each group, we multiplied the delta between the current and the projected future value of the environmental variable associated with the SNP with the regressor of the linear model.

### Ethical approval

All authors declare that animal samples were obtained in compliance with local/national laws in force at the time of sampling. Genotyping data exchange was in accordance with national and international regulations.

## Supplementary Information

Supplementary Information 1.

Supplementary Information 2.

Supplementary Information 3.

Supplementary Information 4.

Supplementary Information 5.

Supplementary Information 6.

Supplementary Information 7.

Supplementary Information 8.

Supplementary Information 9.

Supplementary Information 10.

Supplementary Information 11.

Supplementary Information 12.

Supplementary Information 13.

## Data Availability

The genotyping data for the Italian goat considered in this study are deposited and publicly available on Mendeley Data (DOI: 10.17632/hnd59x6gmg.1; URL: https://data.mendeley.com/datasets/hnd59x6gmg/1).
