# Author Correction: The climatic and genetic heritage of Italian goat breeds with genomic SNP data

**DOI:** 10.1038/s41598-021-98758-3

**Published:** 2021-09-20

**Authors:** Matteo Cortellari, Mario Barbato, Andrea Talenti, Arianna Bionda, Antonello Carta, Roberta Ciampolini, Elena Ciani, Alessandra Crisà, Stefano Frattini, Emiliano Lasagna, Donata Marletta, Salvatore Mastrangelo, Alessio Negro, Ettore Randi, Francesca M. Sarti, Stefano Sartore, Dominga Soglia, Luigi Liotta, Alessandra Stella, Paolo Ajmone‑Marsan, Fabio Pilla, Licia Colli, Paola Crepaldi

**Affiliations:** 1 Dipartimento di Scienze Agrarie e Ambientali – Produzione, Territorio, Agroenergia, Università degli Studi di Milano, Via Celoria 2, 20133 Milan, Italy; 2 Dipartimento di Scienze Animali, della Nutrizione e degli Alimenti and BioDNA Centro di ricerca sulla Biodiversità e sul DNA Antico, Università Cattolica del Sacro Cuore, Via Emilia Parmense 84, 29122 Piacenza, Italy; 3 The Roslin Institute, University of Edinburgh, Easter Bush Campus, Midlothian, EH25 9RG UK; 4 Unità di Ricerca di Genetica e Biotecnologie, Agris Sardegna, 07100 Sassari, Italy; 5 Dipartimento di Scienze Veterinarie, Università di Pisa, Viale delle Piagge 2, 56124 Pisa, Italy; 6 Dipartimento di Bioscienze Biotecnologie e Biofarmaceutica, Università degli Studi di Bari, Via Orabona 4, 70126 Bari, Italy; 7 Consiglio per la ricerca in agricoltura e l’analisi dell’economia agraria (CREA) - Research Centre for Animal Production and Acquaculture, 00015 Monterotondo, Rome, Italy; 8 Department of Agricultural, Food and Environmental Sciences, University of Perugia, 06121 Perugia, Italy; 9 Department of Agriculture, Food and Environment, University of Catania, Via Valdisavoia 5, 95123 Catania, Italy; 10 Dipartimento Scienze Agrarie, Alimentari e Forestali, University of Palermo, 90128 Palermo, Italy; 11 Department of Chemistry and Bioscience, Faculty of Engineering and Science, University of Aalborg, Aalborg, Denmark; 12 Dipartimento di Scienze Veterinarie, Università degli Studi di Torino, largo Braccini 2, 10095 Grugliasco, Italy; 13 Dipartimento di Scienze Veterinarie, University of Messina, Messina, Italy; 14 Institute of Biology and Biotechnology in Agriculture, National Research Council (CNR), Milan, Italy; 15 Dipartimento Agricoltura, Ambiente e Alimenti Universitá degli Studi del Molise, 86100 Campobasso, Italy

Correction to: *Scientific Reports* 10.1038/s41598-021-89900-2, published online 26 May 2021

The Data Availability section in the original version of this Article was omitted.

It now appears as below:

“The genotyping data for the Italian goat considered in this study are deposited and publicly available on Mendeley Data (DOI: 10.17632/hnd59x6gmg.1; URL: https://data.mendeley.com/datasets/hnd59x6gmg/1).”

The original Article has been corrected.

