# Supplementary figures and images for: The climatic and genetic heritage of Italian goat breeds with genomic SNP data
Source: Sci Rep. 2021 May 26;11:10986. doi: 10.1038/s41598-021-89900-2 (PMC8154919; doi:10.1038/s41598-021-89900-2)

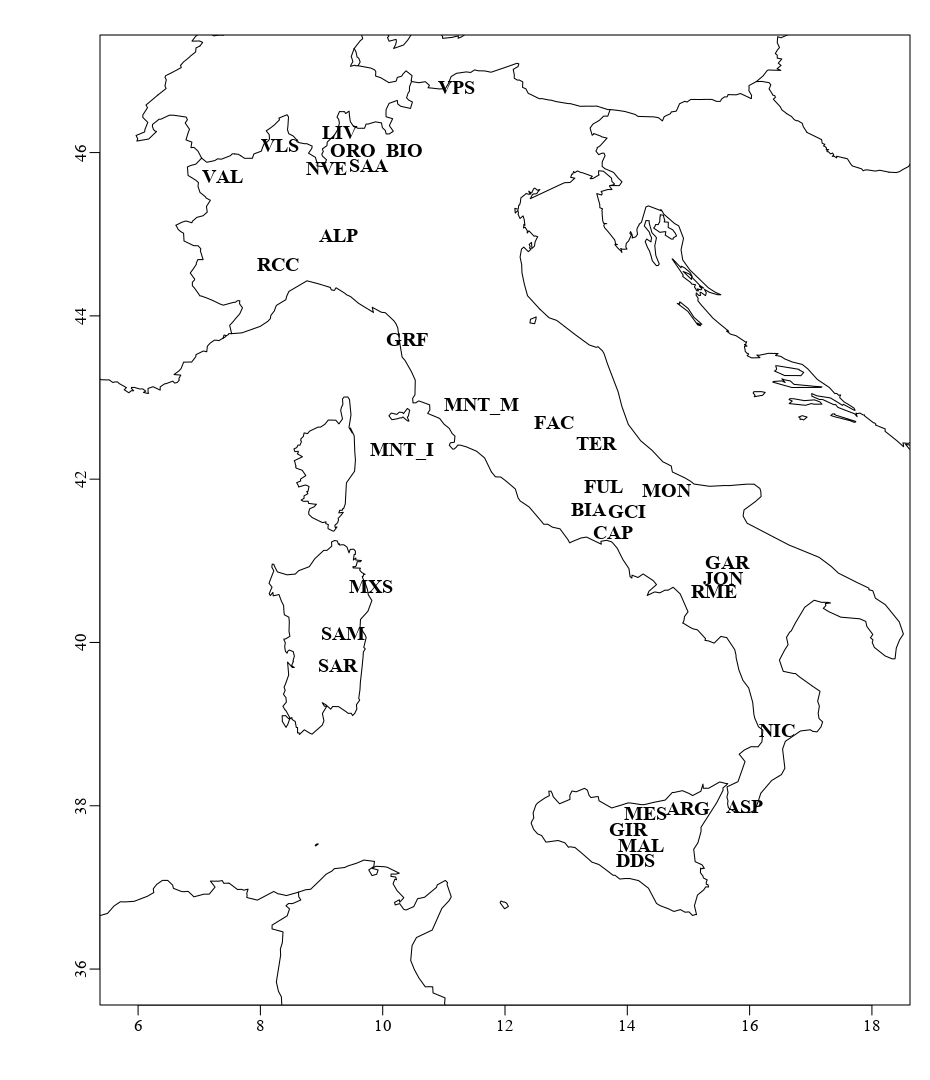

Supplement: Supplementary file 1 — Supplementary Information 1. [file 41598_2021_89900_MOESM1_ESM.tiff]

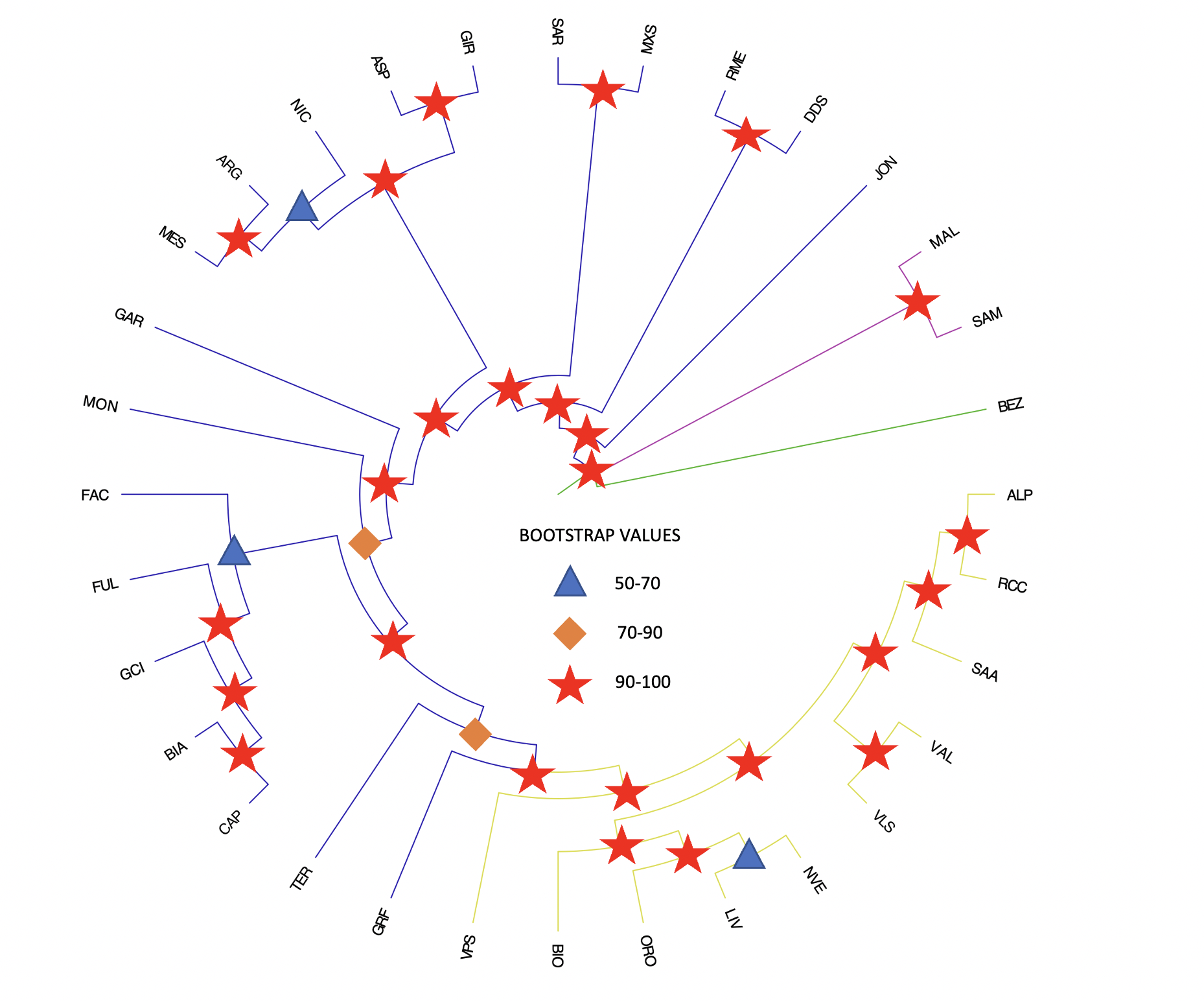

Supplement: Supplementary file 2 — Supplementary Information 2. [file 41598_2021_89900_MOESM2_ESM.png]

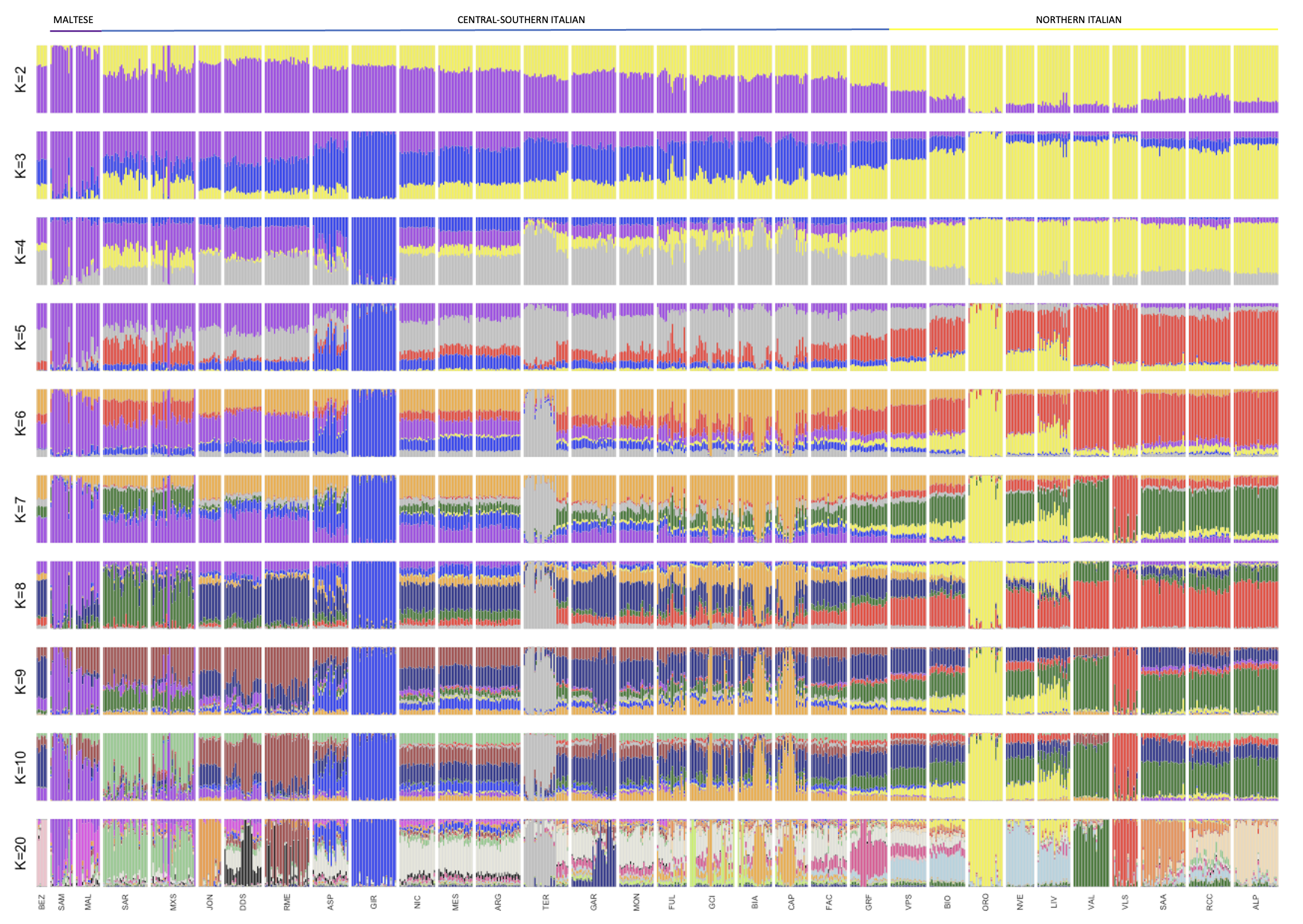

Supplement: Supplementary file 3 — Supplementary Information 3. [file 41598_2021_89900_MOESM3_ESM.png]

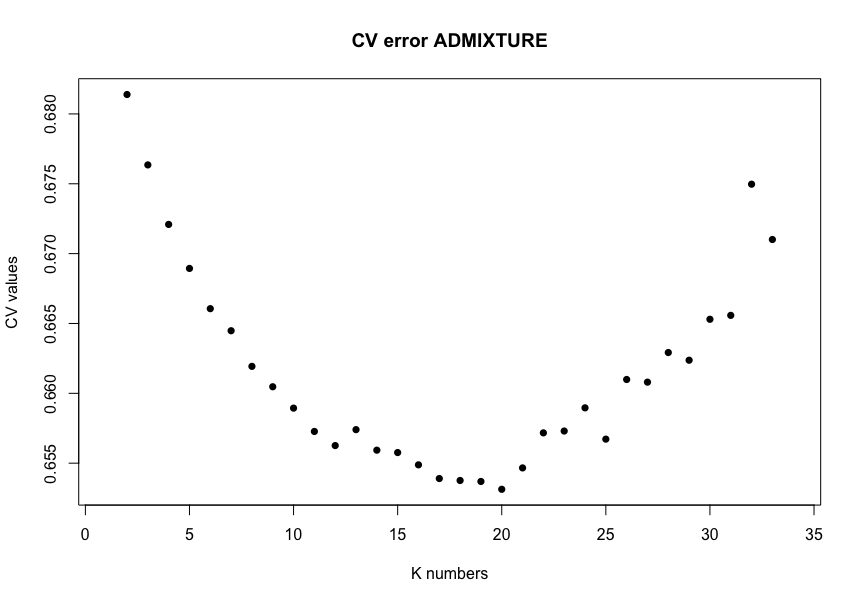

Supplement: Supplementary file 4 — Supplementary Information 4. [file 41598_2021_89900_MOESM4_ESM.tiff]
