# Supplementary material for: The climatic and genetic heritage of Italian goat breeds with genomic SNP data
Source: Sci Rep. 2021 May 26;11:10986. doi: 10.1038/s41598-021-89900-2 (PMC8154919; doi:10.1038/s41598-021-89900-2)
Supplement: Supplementary file 13 — Supplementary Information 13. [file 41598_2021_89900_MOESM13_ESM.docx]

**Supplementary Figures**

**Supplementary Figure 1**: Geographical distribution of the sampling location of the Italian Breeds.

**Supplementary Figure 2**: Bootstrapped phylogenetic tree from Reynolds distances. The plot was generated through the FigTree software ( <http://tree.bio.ed.ac.uk/software/figtree/> )

**Supplementary Figure 3:** ADMIXTURE plot for all K values 2-10 and 20, produced in R using ggplot2 ( <https://ggplot2.tidyverse.org/authors.html> )

**Supplementary Figure 4**: Cross validation error for the different K considered in the ADMIXTURE analyses; the plot was generated through R

**Supplementary Tables**

**Supplementary Table 1**: Samβada significant Results

**Supplementary Table 2**: Genes associated with Samβada significant markers

**Supplementary Table 3**: Correlations among all climatic variables

**Supplementary Table 4** : LFMM significant Results

**Supplementary Table 5**: One-way ANOVA analysis significant results

**Supplementary Table 6**: Results of the linear regression model applied on the significant markers identified by the one way ANOVA analysis

**Supplementary Table 7**: Extended names of the bioclimatic and elevation variables available from the WorldClim database

**Supplementary Table 8**: Koppen-Geiger classification explanation
